# Supplementary material for: Are carbon emissions trading and green financial instruments synergistic? -Comprehensive quantitative research based on content analysis
Source: PLoS One. 2024 Mar 7;19(3):e0298601. doi: 10.1371/journal.pone.0298601 (PMC10919601; doi:10.1371/journal.pone.0298601)
Supplement: S1 Data — (ZIP) [file pone.0298601.s001.zip › Data/地级市Z_F工作报告.zip!/╕ú╜¿/┴·╤╥╩╨/└┤╫╘╣1⁄2╓┌║┼í╛┬φ┐╦╩2╛▌═°í┐_2003.docx]

　　——2003年5月20日在龙岩市第二届人民代表大会第三次会议上

各位代表：

　　现在，我代表市人民政府向大会作政府工作报告，请予审议，并请市政协各位委员和其他列席人员提出意见。

　　一、2002年工作回顾

　　2002年，经过全市人民的共同努力，较好地完成了市二届人大一次会议确定的经济社会发展各项任务。国内生产总值257.86亿元，增长9.5%,超计划0.5个百分点；全社会固定资产投资59.5亿元，增长9.5%，超计划2.5个百分点；财政总收入37.9亿元，增长11.3%，超预算2.1个百分点，其中地方财政收入13.4亿元，同口径增长15%，超预算9个百分点；城镇居民人均可支配收入8543元，增长9.5%；农民人均纯收入3206元，增长4.2%；年末户籍总人口286.8万，人口自然增长率4.6‰。

　　一年来，我们的主要工作是：

　　深化国有企业改革，增长提速，效益提高。深入开展国有企业改革深化年活动，国有工业企业改制面达86.5%，完成国有企业职工身份置换9115人，累计置换率达65%；完成规模以上工业总产值139.6亿元，增长15.9%，增幅为近年之最；完成技改投资12亿元，建成投资百万元以上技改项目15项；效益综合指数153.3，位居全省前列；工业对经济增长的贡献率达43.5%。“佳丽斯”床上用品被评为中国驰名商标和中国名牌产品。

　　实施项目带动战略，投资扩大，后劲增强。进一步明确项目工作的思路、重点，加强向上争取、引进外资、区域协作、激活民资和外事、侨台、旅外乡亲联谊工作，引进区外资金取得较大成绩，对外招商和现有项目延伸拓展取得一定成效。完成或基本完成棉花滩水电站、漳龙高速公路（龙岩段）、省级粮食储备库、龙岩西客站、龙岩卷烟厂易地迁建等一批重点项目建设，赣龙铁路、连城机场改扩建、龙岩大道二期、黄竹坑垃圾处理场等重点项目建设进展顺利。

　　努力发展农村经济，结构调优，农业增效。认真落实农村经济政策，林地林木产权制度改革和土地延包工作顺利推进。农业结构调整取得进展，农作物新品种增加，无公害生产规模扩大。农业产业化步伐加快，农产品加工率达25%，龙头企业发展到110家。劳务开发组织工作加强。防洪减灾体系和气象、水文、防汛预报工作不断完善，抵御了四十年一遇的严重干旱。

　　加快发展第三产业，总量壮大，领域拓宽。加快旅游产业发展，抓好旅游精品建设，加大市场促销力度，接待游客272.8万人次，实现旅游总收入11.7亿元，分别增长25.3%和26.4%。积极扶持交易城兴市旺市，成功举办第二、三届闽西商品交易会。出台鼓励外贸出口政策，支持发展连锁超市、物流配送等现代流通新方式，培育住宅、汽车等新的消费热点。全社会消费品零售总额64.5亿元,增长10.6%。

　　大力加强环境建设，政策优化，服务改进。出台《关于进一步改善投资环境，扩大对外开放的若干规定》和《龙岩市加快企业改革与发展若干规定》。积极推进机构改革和审批制度改革，清理整顿规范性文件，组建行政服务中心，加强编制、人事管理，强化审计监督、效能监察和政府纠风工作。加强科技服务体系建设，建立福建紫金矿业有限公司博士后科研工作站。市人才、劳动力等生产要素市场投入使用，配套环境进一步完善。加强市场监管，整顿和规范市场经济秩序。发挥税收、金融政策的调控引导作用，加强财政收支管理，集中力量支持国企改革、重大项目和开发区建设。

　　全面繁荣社会事业，发展协调，生活改善。认真抓好中小学布局调整、中小学危房改造和初中增班扩容，高考本科上线率名列全省第一。龙岩学院筹建工作进展顺利。群众性文艺活动蓬勃开展。城乡医疗服务水平继续提高，卫生状况有所改善。第十二届省运会取得良好成绩，成功争取到第十三届省运会承办权。全民健身设施建设加强。计生工作连续13年完成省下达控制计划。以粉尘为重点的大气污染治理取得阶段性成效，城市公共交通和绿化、美化、卫生环境有较大改善。国土资源开发与保护得到加强，九龙江和汀江流域生态环境综合整治进展顺利。广泛开展“三下乡”活动。继续抓好小城镇建设和农电改造、改水、改厕、改路等工作。档案、方志、妇女儿童、老龄、助残、民族宗教等工作有新的加强。

　　切实维护群众利益，保障有力，社会稳定。认真做好重点贫困乡村、革命基点村和贫困户的扶贫开发工作，继续实施“五通”和造福工程。重视关心移民群众的生产生活。积极治理“餐桌污染”，严厉查处使用“瘦肉精”等影响食品安全的行为。加强就业和再就业工作，帮助5957名下岗失业人员实现再就业。开展城市社区建设试点。推进保障制度建设，企业职工养老保险、失业保险和城镇职工基本医疗保险参保人数，以及享受城市和农村低保人数有较大增加。加强安全生产监督管理。加大普法宣传，积极开展法律援助。深入开展打黑除恶、反盗抢和查禁“六合彩”等专项行动，积极做好“法轮功”人员的教育转化。加强人防和民兵预备役工作，维护国家安全。

　　回顾过去的一年，在党中央、国务院和省委、省政府及市委的正确领导下，我市经济社会发展全面完成了预期目标。取得这些成绩，是全市上下认真实施“三大重点、五大战略”，加快“八个化”进程，重点推进体制创新、项目带动和新型工业化的结果；是各级人大及各位代表，各级政协及各位委员，各民主党派、工商联，各级工会、共青团、妇联等人民团体大力支持和有效监督的结果。在这里，我谨代表市人民政府，向全市人民，向各民主党派、工商联、无党派人士和人民团体，向离退休的老同志，向中央和省驻岩单位，向驻岩部队、武警部队和公安干警，向所有外来投资者和劳动者，向所有关心支持龙岩的港澳同胞、台湾同胞、海外侨胞和旅外乡亲，表示衷心的感谢！

　　在肯定成绩的同时，我们更清醒地看到加快发展中存在的困难问题，主要是：经济总量小，与沿海的差距还在拉大；生产性项目少，产业结构层次低，工业化进程不快；体制改革比较滞后，利用外资和民间投资规模小；中心城市辐射带动力不强，城镇化水平不高，一些地方基础设施不适应发展要求；城乡就业和农民增收难度加大；县域经济比较落后，不少县乡财政困难；社会治安和安全生产监管还需加强。同时，政府工作中还存在思想不够解放，以改革创新和市场经济手段解决问题、推动发展的办法不多，部分公务员全局意识、责任意识、服务意识和效率意识不强等问题。这些问题，我们将在今后政府工作中努力加以解决。

　　二、今年经济工作安排

　　今年是贯彻党的十六大精神，全面建设闽西小康社会三年打基础的起步之年，也是完成“十五”计划的关键一年。我们要站在全局和战略的高度，克服今年我国突发性非典型肺炎疫情给经济发展所带来的困难，处理好防治工作和经济工作的关系，全面扎实地做好改革发展稳定各项工作，保证国民经济持续快速健康发展。

　　总体思路：以十六大精神为指导，全面贯彻“三个代表”重要思想，紧紧围绕构建山海协作、对内联接、对外开放三条战略通道和全面建设小康社会、走在全国著名革命老区前列的阶段目标，以加快新型工业化和城镇化进程为重点，以实施项目带动战略为载体，以改善发展环境为保障，进一步深入改革，优化资源配置，努力实现有效益、可持续、强后劲基础上的较快增长。

　　主要预期目标：国内生产总值增长9.5%；全社会固定资产投资增长7%；财政总收入增长7%，其中地方级收入增长6%；外贸出口增长20%，实际利用外资增长50%；社会消费品零售总额增长10%；城镇居民人均可支配收入增长5%，农民人均纯收入增长4%；人口自然增长率控制在7.1‰以内。力求各项经济指标能完成得更好些。

　　围绕上述思路和目标，扎实抓好七个方面的工作：

　　（一）大力实施项目带动战略，增强经济发展后劲

　　坚持走“两个并举，两个优先”的路子。坚持资源型与非资源型产业并举，优先发展非资源型产业。继续发展煤、铁、高岭土、稀土等资源深度开发和接续产业，优先发展机械、专用设备制造、轻纺、化工等非资源型产业，鼓励发展新材料、新能源、新环保设备等高新技术产业和农副产品加工等劳动密集型产业。加强产业规划，明确产业走向。制定阶段性发展目标，开发与之配套的阶段性实施项目。坚持基础性与生产性项目并举，优先发展生产性项目。继续抓好交通、电力电网等基础性项目，优先发展符合产业发展方向，能够壮大总量、增加税收、扩大就业、保护环境的生产性项目，培育对区域经济发展具有长效支持作用的增长点、财税点和就业点。

　　严密组织实施“三个一百”项目。逐个项目建立领导挂钩联系制度，成立专门工作小组，配强工作力量。把加强对经济工作的领导落实到抓项目实施上，健全目标管理责任制，实行定期协调会制度。建立主动服务、上门服务和低成本服务机制。加大项目前期经费投入，把政府掌握的资源和生产要素优先向“三个一百”项目倾斜。滚动推进项目实施，形成洽谈一批、签约一批、开工一批、投产一批、见效一批的良好局面。

　　加强重点项目建设。安排重点项目56项（在建重点项目28项，开工新建重点项目12项，重点前期工作项目16项），年度投资30.1亿元。加强组织协调，强化项目进度、质量和投资控制，及时解决资金、征地拆迁、施工环境等问题，确保赣龙铁路、连城机场、交通战备公路、马坑矿业开发等在建重点项目按计划完成投资量。认真做好龙岩至长汀高速公路、龙岩坑口火电厂、重型卡车、高岭土深加工、超纸扩改、龙岩白沙水库电站等项目的前期工作，力争早日动工。

　　创新项目生成机制，加强项目推介工作。围绕新型工业化、城镇化、农业产业化、现代流通、环境保护和社会事业发展，计划部门牵头，各部门、企业和各类专家共同参与，全面开发各类项目源，把项目工作延伸到各行业各领域。努力搭建新的项目转化平台，通过组织参加和举办各类招商会、项目推介会、信息发布会，积极向上、向外争取项目，主动与大企业对接寻找项目，促进项目转化实施。

　　（二）加快工业化进程，增强工业经济的主导作用

　　加强对企业的支持与服务。突出抓好重点工业企业，及时掌握生产经营动态，建立煤炭、电力、运力、原辅材料、资金等主要生产要素的协调机制。扶持企业加强质量体系建设，开展质量认证工作，争创名牌，开拓市场。进一步加强银企协作，健全中小企业担保服务体系，促进企业更快发展。

　　加快工业结构调整。抓好烟草、冶金、建材、机械、纺织等重点产业发展，做大做强龙烟、龙钢、龙工、龙净、紫矿、超纸等骨干企业和“佳丽斯”床上用品、“七匹狼”卷烟、“龙工”装载机、“龙净”环保设备等名牌产品，延伸产业链，做大产业群。推进重点技改项目实施，加强中小企业的技术改造，鼓励增加技改投入。扶持重点科技项目的研究、开发和应用，加强企业技术中心建设，促进产学研结合。

　　推进国有企业改革。以改强、改大、改活国有企业为目标，加快建立现代企业制度，推进“两个置换”，基本完成国有工业企业改制，职工身份置换面达80%以上。认真落实支持国有企业改革与发展的各项政策，加大出让、减持优质国有股力度，加强与各金融资产管理公司沟通协调，积极吸引外资、民资参与国有企业改革，推动股份公司的设立和争取上市工作。进一步深化企业劳动、人事、分配制度改革，强化决策、财务、成本、质量管理。

　　加快工业园区建设。加强产业布局和功能定位，加快龙岩经济技术开发区等工业园区建设。实行公司化的市场开发体制，落实园区发展的优惠政策，放宽入区项目的管理审批权限，建立项目全程代办的服务体系，加快园区内水、电、路等基础设施建设，营造局部招商优势。

　　（三）全面发展农业和农村经济，努力促进农民增收

　　深化农村改革，创新工作机制。深入实施《福建省乡镇工作纲要》，稳步推进农村税费改革试点和农村土地承包经营权流转，深化林地林木产权制度、粮食购销市场化和小型水利设施产权改革，建立多元化的投入机制。继续加快农业新品种、新技术的引进、研制、试验示范、推广，建设一批现代农业高新技术示范园区。推进农业服务、流通体制改革。落实农村减负政策，加强学校、医院、建房审批等收费监管，切实减轻农民负担。加强县乡财政债务管理，落实减债责任制。

　　加快结构调整，提高农业综合效益。加快发展优质、高效经济作物，积极发展瘦肉型猪、河田鸡、白鸭、槐猪、肉兔、奶牛等重点优质畜禽和天然优质淡水鱼类，大力发展无公害农产品、绿色食品和有机食品。加强农业基础设施建设，完善防灾减灾体系，加快重点农产品标准体系、农产品质量标准检验检测体系、动植物疫病防疫体系建设。推进以长汀为重点的水土流失治理，提高农业生产综合保障能力。

　　推进产业化经营，提高生产组织化程度。围绕八大主导产业和五大重点特色产品，着力培育一批产业关联度大、装备水平高、竞争力和带动力强的龙头企业。进一步提升地瓜干、花生、红柿、杭梅等大宗农产品加工能力，培育果蔬生产、保鲜、加工产业群；鼓励个私造林，大力发展短周期工业原料林、丰产竹林和食用菌林，培育木竹生产、加工产业群；实施瘦肉型猪产业化二期项目，培育畜牧养殖、加工、饲料、兽药产业群。完善市、县、乡三级农产品营销信息网络，推行“公司加农户、中介加农户、科技流通服务一体化”等形式，发展规模经营。

　　加强劳务开发，提高非农收入比重。加强职业技能培训和就业信息指导，加快发展劳务中介组织，规范中介行为。通过发展劳动密集型产业、推进城镇化进程、实施“回乡创业工程”等多种渠道，加快农村劳动力向非农产业和城镇转移，力争全年新增转移农村劳动力7万人以上，其中劳务输出4万人。

　　（四）大力发展第三产业，培育新的经济增长点

　　大力发展现代物流业。积极吸引国内外先进物流企业投资，继续鼓励发展连锁经营、物流配送和电子商务。改造提升城乡商品交易市场，推行市区“农改超”。实施闽西现代物流园区项目，发展城市交通运输和货物中转业务。

　　加快发展旅游生态经济。加大宣传促销力度，拓展海内外市场。推进区域协作，整合旅游资源，加强“红色之旅”、“客家之旅”、“绿色之旅”等特色精品线路建设。积极做好连城争创“全国首批优秀旅游县”，永定洪坑土楼民俗文化村、龙崆洞、培田古民居、云顶茶园、古田会址旅游区“创A”和梅花山申报国家级生态旅游示范区、紫金山申报省级工业旅游示范区工作。建设古田生态文化经济开发区。加快开发各类旅游消费品，逐步形成配套的旅游产业体系。

　　积极发展其他服务业。重视运用现代经营方式和先进技术，改造交通运输、餐饮、公用事业等传统服务业。积极发展房地产业，加快建设面向中低收入群体的经济适用房。鼓励发展物业管理、社区服务、教育培训、文化、体育等需求潜力大的行业，大力发展金融、信息、保险以及会计、咨询、法律服务、科技服务等中介服务业，不断壮大第三产业总量。

　　（五）做大做强中心城市，积极推动县域经济发展

　　壮大城市经济。夯实产业基础。加快黄邦山经济技术开发区和新罗区张白土高新技术园区的发展，把两区外围基础设施作为城建重点，为扩大城市规模、壮大经济总量提供载体。抓好交易城二期项目，继续支持交易城兴市旺市。大力发展城市商贸、交通、旅游、房地产业，增强城市产业配套能力。

　　完善城市功能。继续完善城市路网、地下管网、公共交通网络、停车场及其它公共设施。把路网建设与旧城改造和带动新区开发结合起来，抓好龙岩大道二期及两侧整体拆迁、双龙路、登高西路等项目，尽快打通南北通道和东西通道，构建中心城市路网骨架。加快黄竹坑垃圾处理场建设，优化垃圾中转站布局，提高城区污水集中处理覆盖面。

　　提高管理水平。加强城市规划。开展中心城市总体规划修编工作，抓好新区设计、人民广场周边5平方公里城市概念规划和3平方公里的修建性详细规划。认真落实《福建省城市社区建设纲要》，进一步健全管理体制，落实管理责任。加快实施相对集中行政处罚权工作，改革和完善执法体制，推进依法管理城市。

　　加快发展县域经济。认真落实加快县域经济发展的政策措施，营造良好环境。扩大县级经济管理权限，增强县域经济发展的主动权。全面推进工业化、城镇化和农业产业化，大力发展非公有制经济。加强规划引导，鼓励和扶持发展具有比较优势的特色支柱产业，培植主体、骨干财源。继续加强农村路网、电网等基础设施建设。

　　（六）加快开放步伐，大力发展非公有制经济

　　全力进行招商引资。重点在工业、农业、城市建设与产业经营、卫生与教育、服务贸易、旅游开发和能源开发等领域，推行厂房、土地、股权、矿业权及无形资产招商。有针对性地实行小规模招商、专业招商、委托招商、代理招商和网上招商，加快构建政府、企业和中介机构三位一体的招商引资新平台。继续实行招商引资分级分类跟踪落实责任制和部门责任制，促进项目签约、报批、到资。继续实行出口奖励政策，健全外贸出口服务体系，促进对外贸易增长。

　　大力激活民资。进一步放宽市场准入，降低市场门槛，把优势资源开发、优势国企嫁接、优势领域的发展面向非公经济放开，促进产业链的上下游延伸和企业间的配套协作。以财税贡献、就业、投资规模、科技含量等指标作为享受优惠政策的标准，扶持非公经济发展。加强服务，着力解决非公企业融资难问题，依法保护非公经济的合法权益，促进非公企业做大做强。

　　加强区域协作。健全完善市、县经济协作工作机制，巩固发展闽西南五市、闽粤赣边和闽粤赣十三市三个协作区的经济合作，主动接轨沿海，融入闽东南经济区，不断拓展与广州、深圳、上海和其他地区的合作。

　　（七）推进资本运作和资源整合，加强有效调控

　　加大城市经营力度。建立政府主导型的土地收储、出让制度，合理调控房地产用地总量，实现城市经营性土地价值最大化。租赁、公开拍卖、有偿转让城市供水、环卫维护、园林绿化、户外广告经营权、公交线路和出租车经营权以及道路、桥梁、广场、雕塑的冠名权，加快自来水、污水和垃圾处理产业化步伐。进一步剥离公园、公厕、停车场等市政公用设施的所有权、经营权和养护管理权。采取租、售并举方式，抓紧处理闲置房产，加快回笼建设资金。

　　盘活国有资产。依法加强国有资产管理。完善交通基础设施建设管理运营体制，盘活公路设施固定资产，增强增量资金投入的能力。优化配置教育、卫生、文化等社会事业资源，鼓励社会资金参与建设经营，实现部分产业化。着手行政事业单位资产清查，整合利用闲置资产。做好矿产、水力、旅游等资源开发的规划、论证，逐步推向市场。

　　集中财力办大事。坚持依法治税、依法理财。合理配置财力资源，优化支出结构，在优先保证工资发放、社会保障和社会稳定支出的基础上，把财政工作的立足点放在培育新经济增长点上，重点支持项目建设、企业改革、结构调整等扶持经济发展的支出。加强支出管理，强化预算约束，继续做好会计核算中心和国库统一发放工资的扩面工作，严格实行预算外资金收支两条线制度。加强审计、财政监督，严肃财经纪律。强化减债也是政绩观念，积极防范和化解财政风险。

　　加强金融支持服务。重视“贷款难，难贷款”问题，以项目为纽带，密切银企、银农、银贸联系合作，促进银行贷款的适度增长。抓好农村信用社改革，加强信贷支农。坚决制止企业逃废金融债务行为，加强信用体系建设，稳定地方金融秩序。

　　三、繁荣各项社会事业，维护和发展群众根本利益

　　扎实抓好“非典”防治工作。按照不发生二代感染、不发生医护人员感染、不发生死亡病例的“三不”目标要求，加强领导，落实责任，执行政策，依靠科学，依法防治，健全完善群防群治的网络机制、救助机制和安全有效的治疗机制。认真落实防治工作的属地管理原则，加强各级各部门履行防治职责的纪律约束。配备健全规范的医疗场地、设备、器械和医护人员，提高应急处置能力。加大宣传工作力度，广泛开展爱国卫生活动，不断推进疾病预防控制体系建设，确保人民群众的身体健康和生命安全。

　　加强就业和再就业工作。落实政府促进就业和再就业责任制，加强下岗失业人员的转岗培训，重点做好“4050”人员等就业困难群体的再就业援助工作，积极引导下岗职工通过市场就业、自主择业。加强市、县、乡三级人才市场、劳动力市场和就业服务网络体系建设，充分发挥工会、街道办事处、居委会等就业服务功能，健全机制，规范服务。

　　落实完善社会保障制度。增加社会保障投入。认真做好“两个确保”和“三条保障线”的衔接，确保国有企业下岗职工基本生活费和企业离退休人员养老金按时足额发放。完善城市和农村居民最低生活保障线制度，加强失业、养老及农村社会保险的扩面征缴，扩大医保覆盖面。

　　继续加强老区建设和扶贫工作。加大小额信贷扶贫力度。抓好扶贫开发重点村、革命基点村、少数民族村的经济发展，实施造福工程和自然村通路、通水、通有线电视、通电话工程。加强重点工程建设移民安置及后续工作，不断改善移民群众的生产生活条件。

　　全面落实三大国策。坚持计生工作党政一把手亲自抓、负总责，加强依法管理、综合治理、政策推动和分类指导，狠抓流动人口管理，推动村自管理和村民自治，努力稳定低生育水平。认真落实环境保护目标责任制，扎实推进生态市建设。巩固中心城区水泥企业粉尘治理成果，按计划推进高速公路、国省道沿线及重要景区水泥和其它主要排污企业的污染治理；继续抓好九龙江北溪、汀江、上杭紫金山、新罗中甲矿区和养殖业等重点流域、重点矿区、重点产业的污染整治。加强国土资源保护与开发，继续深入开展土地市场和矿产资源管理秩序治理整顿，制止耕地抛荒，加强土地整理，保障重点项目建设用地需求，实现耕地总量动态平衡。

　　加快发展社会各项事业。加强科技研究、开发和应用，加快构建科技创新、普及服务体系。巩固提高教育“两基”成果，实施“双高普九”规划，推进中小学危房改造和薄弱学校改造，完成初中增班任务。抓好龙岩学院筹建，确保首期工程按期建成投入使用。积极探索新型农村合作医疗制度，健全农村和社区卫生服务网络，改善医疗卫生条件。加快龙岩图书馆及县乡村文化基础设施建设，继续实施农村广电入户工程，规范和发展文化市场。实施“全民健身工程”，以承办2006年第十三届省运会和第七届老年运动会为契机，加快体育场馆的总体规划建设，培养体育竞技人才。发展老龄、妇女儿童、残疾人保障事业，继续做好方志、档案、侨务、民族宗教、文物保护等各项工作。认真组织实施为民办实事项目。

　　加强精神文明和民主法制建设。深入实施《公民道德建设实施纲要》，推进社会公德、职业道德和家庭美德建设。深入开展群众性精神文明创建活动，丰富群众文化生活。加强人防、民兵预备役工作，争创全国“双拥”模范城。加强城乡卫生整治和管理，着力改变“脏、乱、差”现象。加快农村殡葬改革，反对封建迷信活动。推进民主法制建设，依法全面完成村级换届选举。加强村级财务管理，完善村委会民主决策、民主管理和民主监督机制。继续实施普法教育，增强全社会的法律意识。

　　大力维护社会稳定。积极开展基层创安活动，坚决落实社会治安综合治理“一票否决”制。认真做好群众来信来访工作，加大民事纠纷的排查调处力度，加强法律援助，把矛盾化解在基层和萌芽状态。继续深化严打整治斗争，严厉打击各类刑事犯罪、经济犯罪、“六合彩”骗赌活动和“法轮功”邪教活动。落实安全生产责任制和重大安全事故责任追究制，深入开展煤矿、烟花爆竹、道路交通、非煤矿山、公众集聚场所、危险化学品、学校、水上交通、锅炉压力容器、建筑业、旅游业等安全专项整治，加强重大节日、重要活动、重点部位的安全防范工作。

　　四、建设廉洁、勤政、务实、高效、诚信的服务型政府

　　全面建设小康社会需要一个能够全面履行职责，高效服务企业和社会的服务型、责任型政府。各级政府要始终坚持“两个务必”，在市委领导下，通过自觉接受人大及其常委会的法律监督和工作监督，接受政协、民主党派，以及新闻舆论和社会各界的民主监督，进一步增强加强自身建设的自觉性和责任感；继续加强与政协、民主党派、无党派人士、工商联和工会、共青团、妇联等人民团体的联系沟通，进一步提高政府科学决策和履行职责的能力和水平，努力把各级政府建设成为廉洁、勤政、务实、高效、诚信的服务型政府。

　　加强廉政建设，维护社会公正。廉洁是政府的第一形象，是公正和效率的基本保证。要进一步深化政务公开，建立政府重大事项公示制度和重大决策听证制度；严格遵循政府采购、经营性土地出让招标、建设工程招投标、产权交易等各项法律、制度、规定，维护公平、公正的市场竞争秩序；深入开展反腐纠风工作，严厉打击政府管理行为和管理过程中以权谋私、权钱交易，破坏正常经济秩序、损害社会诚信、影响政府形象的人和事。

　　加强勤政建设，提高服务效率。各级政府和政府各部门要围绕发展抓服务，围绕项目抓服务，把企业和投资者满意不满意作为评价政府工作成效的重要标准，只要对发展有利，对全局有利，对造福闽西百姓有利，就要敢于拍板，敢于负责。坚持实事求是，把对上负责与对下负责结合起来，注重研究政策，善于协调关系，具体问题具体分析，具体项目具体对待，特殊项目特殊处理。以减少审批、简化审批和推行告知承诺制为重点，深化审批制度改革。

　　加强作风建设，发展群众利益。进一步拓宽与人民群众的联系渠道，注意解决群众和投资者关注的热点、难点问题。坚决反对官僚主义和形式主义，杜绝“形象工程”、“政绩工程”和“文山会海”，严格控制达标升级、评比检查活动，努力降低行政成本。坚持诚实守信、讲到做到，兑现对社会、对人民的承诺，取信于民。

　　加强机制建设，促进依法行政。推进机构、编制、人事管理改革，增强政府经济调节、市场监管、社会管理和公共服务职能。推进政府法制建设，理顺行政执法体制，规范执法主体，落实执法责任，强化执法监督，提高执法水平。加强民主决策制度建设，促进决策科学化、规范化、制度化。

　　建设廉洁、勤政、务实、高效、诚信的服务型政府，必须落实到具体的工作中，体现在构筑良好的发展环境上。必须有一套适应形势，符合实际，能吸引市内外投资者，激发社会创业热情的鼓励政策；有一套便捷、高效、低费、周到的服务机制；有一个规范有序、公开透明、竞争平等的市场环境。各级政府、政府各部门和全体公务员要围绕发展，一手抓项目，一手抓环境，立足岗位，从我做起，敢于直面发展的难题，善于破解发展的障碍。要严肃工作纪律，对损害经济发展软环境行为坚决依纪处理。

　　各位代表，今年以来我市经济发展势头良好。1-4月,全市完成规模以上工业总产值51亿元，增长22.2%；固定资产投资11.88亿元，增长55.3%；财政总收入14.15亿元，增长12.6%，其中地方级收入5.47亿元，增长28.1%；社会消费品零售总额23.74亿元，增长19.5%。此外，农业生产、外经外贸等都完成了序时任务。

　　各位代表，闽西的发展正处于一个十分重要而关键的时期。让我们紧密团结在以胡锦涛为总书记的党中央周围，在市委的领导下，深入学习贯彻党的十六大精神，认真实践“三个代表”重要思想，万众一心，奋发图强，脚踏实地，勤奋工作，努力夺取防治非典型肺炎的胜利，圆满完成今年经济社会发展的各项任务，为加快建设闽西全面小康社会而奋斗！
